# Early Brain Loss in Circuits Affected by Alzheimer’s Disease is Predicted by Fornix Microstructure but may be Independent of Gray Matter

**DOI:** 10.3389/fnagi.2014.00106

**Published:** 2014-05-28

**Authors:** Evan Fletcher, Owen Carmichael, Ofer Pasternak, Klaus H. Maier-Hein, Charles DeCarli

**Affiliations:** ^1^IDeA Laboratory, Department of Neurology, University of California Davis, Davis, CA, USA; ^2^Department of Computer Science, University of California Davis, Davis, CA, USA; ^3^Departments of Psychiatry and Radiology, Harvard University, Cambridge, MA, USA; ^4^Medical Image Computing Group, German Cancer Research Center (DKFZ), Heidelberg, Germany

**Keywords:** fornix diffusivity, longitudinal brain change, limbic circuit, default mode network, normal cognition

## Abstract

In a cohort of community-recruited elderly subjects with normal cognition at initial evaluation, we found that baseline fornix white matter (WM) microstructure was significantly correlated with early volumetric longitudinal tissue change across a region of interest (called fornix significant ROI, fSROI), which overlaps circuits known to be selectively vulnerable to Alzheimer’s dementia pathology. Other WM and gray matter regions had much weaker or non-existent associations with longitudinal tissue change. Tissue loss in fSROI was in turn a significant factor in a survival model of cognitive decline, as was baseline fornix microstructure. These findings suggest that WM deterioration in the fornix and tissue loss in fSROI may be the early beginnings of posterior limbic circuit and default mode network degeneration. We also found that gray matter baseline volumes in the entorhinal cortex and hippocampus predicted cognitive decline in survival models. But since GM regions did not also significantly predict brain-tissue loss, our results may imply a view in which early, prodromal deterioration appears as two quasi independent processes in white and gray matter regions of the limbic circuit crucial to memory.

## Introduction

Recent work has shown that the fornix (Oishi et al., 2012; Fletcher et al., 2013a) and other memory-related white matter (WM) tracts (Zhuang et al., 2012, 2013) are sensitive predictors of conversion from normal cognition to mild cognitive impairment (MCI) or Alzheimer’s dementia. In these findings, the loss of WM volume or microstructural integrity, in key structures, appears to be measurable before gray matter differences. Similarly, in groups of subjects with and without subjective memory complaints, but scoring normally on all cognitive tests – thus encompassing very subtle differences – WM diffusivity of the posterior cingulate, retrosplenial cortex (RSC), and precuneus (Selnes et al., 2012) and parahippocampal WM (Wang et al., 2012) was significantly different for those with memory complaints, while gray matter thickness was not. Recent results such as these have been taken to suggest that early WM deterioration may be at least partially separate (Agosta et al., 2011; Selnes et al., 2012) from the β-amyloid and tau pathologies of gray matter, whose evolution is known to characterize Alzheimer’s disease (AD) (Braak and Braak, 1996), and have even raised the question whether Alzheimer’s should be considered a disease of the WM (Sachdev et al., 2013).

On the other hand, the progression of β-amyloid and tau pathologies has long been established (Braak and Braak, 1996) and remains the dominant paradigm of AD modeling (Jack et al., 2010; Sperling et al., 2011; Jack and Holtzman, 2013). In these models, gray matter changes due to an “amyloid cascade” and the tauopathy driven by it (Jack and Holtzman, 2013) underly brain atrophy and deterioration. The sequence of events consists of GM atrophy followed by WM disconnection and regional hypometabolism (Villain et al., 2008, 2010). This deterioration is best viewed as a network disconnection syndrome (Reid and Evans, 2013) in which specific networks – the Papez memory circuit (Villain et al., 2008; Acosta-Cabronero et al., 2010), also known as the limbic circuit (Nestor et al., 2003), together with the default mode network (DMN) (Greicius et al., 2004) – are selectively affected (Seeley et al., 2009). In recent biomarker models of AD progression (Jack and Holtzman, 2013), tauopathy may or may not arise before the amyloid cascade but increasing levels of β-amyloid, perhaps due to inadequate clearance from the brain, drive the trajectory of brain deterioration. Thus according to the “amyloid cascade hypothesis” (Jack and Holtzman, 2013), tauopathy is separate from amyloidopathy but dependent on it. When tauopathy progresses, it follows the well-known stereotypical pattern starting with the brainstem and transentorhinal cortex (Braak and Braak, 1996; Jack and Holtzman, 2013). The biomarker models (Jack et al., 2010; Jack and Holtzman, 2013) therefore picture an ordering of amyloid and tau pathologies followed eventually by macroscopic brain changes and measurable declines in memory and clinical function.

Thus, there may be an apparent contrast between amyloid models and recent results, among preclinical subjects, suggesting the importance of WM differences as early markers of decline. This has raised the question whether the biomarker sequence of Jack and Holtzman (2013) should be modified to include measurable WM deterioration as one of the earliest events (Sachdev et al., 2013). It would therefore be useful to clarify the extent to which baseline white and gray matter measurements, in the prodromal phase preceding MCI and AD, are associated with longitudinal changes in the brain. This could help evaluate recent suggestions about the usefulness of key WM tracts as early biomarkers (Zhuang et al., 2012; Fletcher et al., 2013a; Sachdev et al., 2013) to supplement the CSF and brain biomarkers (Jack et al., 2010; Jack and Holtzman, 2013) of the amyloid models. And by comparing the sequencing of gray and WM changes, we may gain clues about whether white and gray matter processes are related or separate, thus advancing our knowledge of the ways in which the brain starts to change prior to measurable cognitive decline.

In this article, we investigate whether baseline measurements of cortical gray matter volume and WM microstructure, in regions of interests (ROIs) known to be associated with cognitive decline, are also predictors of early longitudinal brain changes in a cohort of clinically normal subjects. Our GM ROIs are the hippocampi, entorhinal cortical gray, and gray matter in the RSC and posterior cingulate – known sites of early and later AD pathology – as well as the anterior cingulate GM for comparison. WM ROIs include the fornix, which has been shown to be a predictor of earliest cognitive decline, along with the splenium and anterior and posterior cingulum, whose integrity is diminished in MCI or AD (Zhang et al., 2007; Mielke et al., 2009; Acosta-Cabronero et al., 2010). We also test the genu and corticospinal tract (CST) as WM tract controls. Our comparison of baseline WM with longitudinal change has some precedent. It is similar to the approach in an earlier study of middle-aged normal subjects (Ly et al., 2013), which found that baseline fractional anisotropy (FA) measurements in entorhinal WM, corpus callosum genu, and splenium were associated with tissue loss in the superior longitudinal fasciculus (SLF), corona radiata, and other regions. This indicated that baseline WM measurements may be predictive of WM tissue change even in relatively young subjects – 15 years before the lower age cutoff of our normal elderly cohort – and suggested that WM microstructure and tissue loss should be included in models of aging.

As in that study, we did not suppose an *a priori* hypothesis about *which* specific brain regions may be associated with baseline cortical GM thickness or microstructural features in the WM ROIs. But we did test the hypothesis that tissue loss in *some* brain area is associated with baseline measurements in one or more of these ROIs. And we analyzed these findings to attempt inferences about the nature and timing of brain processes preceding cognitive change in elderly normals.

## Materials and Methods

### Participants

Subjects for this study consisted of cognitively normal individuals recruited into the Longitudinal Cohort of the Alzheimer’s Disease Center of the University of California Davis (UCD ADC). Participants were recruited through community outreach using methods designed to enhance ethnic diversity (Hinton et al., 2010). All subjects provided informed consent before participating in this study.

### Clinical evaluation

Each participant received multidisciplinary clinical evaluations at the UCD ADC at baseline and at approximately annual follow-up examinations. Evaluations included detailed medical history, with physical and neurological examinations. Diagnosis of cognitive syndromes – MCI or AD – was made according to standardized criteria (Mungas et al., 2011) by a consensus conference of clinicians. The clinical dementia rating sum of boxes score (CDRSum) (Morris, 1997) was assessed as a measure of clinically relevant functional impairment. Clinical “conversion” from normal cognition occurred when a subject with normal cognition at baseline was diagnosed as MCI or AD at a follow-up visit. The time to conversion was measured as the time from baseline to date of conversion.

### Magnetic resonance imaging

Each subject received at least two structural T1-weighted MRI scans, with the first one close to the date of the baseline clinical evaluation, as well as a diffusion MRI sequence at the same date as the first scan. All images were acquired at the University of California Davis, Imaging Research Center. The T1-weighted spoiled gradient recalled echo acquisition had the following parameters: TR 9.1 ms, flip angle 15°, field of view 24 cm, slice thickness 1.5 mm, and field strength 1.5 T. The 2D axial–oblique single-shot spin-echo planar imaging diffusion sequence had the following parameters: TE 9.4 ms, TR 8000 ms, flip angle 90°, field of view 24 cm, and slice thickness 5 mm. In-plane voxel dimensions were 1.875 mm in each direction. The *B* value was 1000 s/mm^2^ with six gradient directions collected four times each (two times each in the plus and minus directions), plus two B0 images. In order to perform group statistical analysis, all subject T1 images at baseline were non-linearly deformed to a minimal deformation template (MDT) (Kochunov et al., 2001) using linear and non-linear B-spline transformation parameters (Rueckert et al., 2006). Diffusion images were corrected for eddy currents in native space using the FSL toolbox (Jenkinson et al., 2012). To account for the presence of extracellular free water contamination as partial voluming in the diffusion images, diffusion tensors were estimated by the free water elimination (FWE) algorithm (Pasternak et al., 2009). This algorithm estimates at each voxel the contributions to apparent diffusivity of separate tissue and free water compartments, returning both a voxel map of free water fractional volume (from 0 to 100%) and estimated diffusion tensors that represent tissue without free water. Scalar measures of FWE-corrected FA were calculated from the diffusion tensors. In addition to free water corrected maps, we previously calculated regular (uncorrected) FA maps that were warped to a template for group analysis. An FA–MDT was constructed as the average of 69 cognitively normal subject FA images, each linearly aligned to its corresponding native T1 image and then transformed to MDT space using the transformation parameters generated for its T1 image, after visual verification that the linear alignment between native FA and T1 was accurate. For the current study, native subject uncorrected FA baseline images were separately linearly aligned and B-spline deformed directly to the FA–MDT image that was already coincident with our MDT. The transformation parameters deforming native FA to FA–MDT were then used to deform individual subject diffusivity images (FWE-corrected FA and Free water maps) into template space. In the remainder of this article, we refer to all FWE-corrected FA maps simply as FA.

### Longitudinal change analysis

Each normal subject received a T1-weighted structural scan at a date close to the baseline clinical evaluation and second scan at least a year later. Localized voxelwise longitudinal change between the two T1 images was computed using a tensor-based morphometry (TBM) algorithm designed to enhance sensitivity and specificity by incorporating knowledge of likely tissue boundary locations (Fletcher et al., 2013b). Brain change was quantified using log-Jacobians derived at each voxel from the TBM deformation field. In this analysis, the determinant of the 3 × 3 derivative matrix of the deformation field computes the local volume change factor indicated by the deformation. Determinants between 0 and 1 indicate volume shrinkage or loss, while determinants >1 indicate expansion. Thus, Jacobians represent multiplicative proportional volume changes from baseline. Performing a log transform then gives a distribution of values symmetric about 0, with negative logs indicating contraction, positive logs indicating expansion, and 0 indicating no volume change (Hua et al., 2008; Fletcher et al., 2013b). These will be referred to as the log-Jacobians. In longitudinal brain mapping of our elderly cohorts, log-Jacobians of CSF spaces like the ventricles and sulci are generally positive, while those located in tissue are negative, indicating patterns of tissue loss and CSF expansion. To perform group statistical analysis, native space log-Jacobian images were deformed into template space using the linear and B-spline parameters previously computed for native T1 images as described above. For valid group comparison, log-Jacobian images in template space were normalized to represent change over a 2-year period. This was done by multiplying each Jacobian image by a factor consisting of the ratio 2.0/ΔT, where ΔT was the interscan time interval for that subject’s two T1 MRI scans. This normalization is justified by the assumption, verified empirically on sample subjects having several scan times, that brain-tissue change is roughly log-linear over time.

### Regions of interest

Regions of interest were used to evaluate the correlations of baseline measurements in both white and gray matter with longitudinal changes. For WM measurements, template MDT space ROIs were used to examine the association of mean FA with voxelwise brain changes. For the fornix body we used an ROI generated in-house by an experienced neurologist, and previously used in publications from our lab (Lee et al., 2012; Fletcher et al., 2013a). The genu and splenium of the corpus callosum and the anterior and posterior cingulum ROIs were generated by the same neurologist. We tested baseline mean FA values in these regions for the existence of significant clusters of associated tissue change as measured by our log-Jacobians. We also ascertained which relevant WM structures overlapped our clusters of significantly associated log-Jacobian change. We calculated overlaps of the Jacobian clusters with our thalamus, splenium, and cingulum ROIs, Brodmann areas (BA) also generated in-house, together with ROIs in the Johns Hopkins WM atlas (Zhang et al., 2010), all warped to our own MDT. For GM measurements, ROI GM volume was ascertained in each subject’s native space. BA template MDT ROIs were inversely transformed to native space structural T1 images via a process we have used in previous publications (Fletcher et al., 2013a,b). GM volumes were computed from these ROIs overlaid over the native space four-tissue segmented image. Tissue segmentation in the native T1 images was accomplished using a Bayesian maximal-likelihood algorithm enhanced for sensitivity at tissue boundaries (Fletcher et al., 2012). Hippocampal volumes were computed in native space by hand tracing methods that have been described previously (Carmichael et al., 2010; Lee et al., 2012). To test the hypothesis that baseline GM measurements were associated with longitudinal brain change, we used the hippocampi and BA ROIs that are known to be implicated in GM loss during the trajectory of cognitive decline – BA 28 and 34 (the ventral and dorsal bilateral entorhinal cortices).

### Statistical analysis

To assess the correlation of baseline ROI GM or WM measurements with longitudinal change, we used single linear regression models of ROI mean FA values or GM volumes and voxelwise log-Jacobian values, computing the Student’s *t*-value at each voxel for the slope of the regression line. In these regressions, the slope of the regressions at brain-tissue voxels will generally be positive for ROI FA values, corresponding to the association between lower FA values (putative markers of impaired ROI microstructure) and more negative log-Jacobians (indicating more severe brain-tissue loss). Conversely, higher ROI FA values should correspond to less severe brain-tissue loss, computed by negative log-Jacobians of smaller magnitude. Similarly for GM ROIs, the associations should be positive in brain-tissue areas, corresponding to a smaller magnitude of local tissue loss associated with greater baseline GM volume. The *t*-value clusters were corrected for multiple comparisons (Nichols and Holmes, 2001). We did this by non-parametric permutation testing over 1000 iterations. We tested for size of contiguous voxel clusters all having regression *t*-value >3.5 for both the FA values and the GM volumes. Clusters with sizes in the top 95th percentile over all iterations were taken as regions of significant association (*p* < 0.05) between ROI FA or GM and longitudinal brain change.

To evaluate the significance of brain factors for cognitive conversion, we constructed Cox proportional hazards models (Cox, 1972). Each model contained demographic factors of age, gender, education, and ethnicity as covariates, together with a single brain measurement. A brain measurement was a significant factor in cognitive conversion if the probability of having a larger χ^2^ than the one in the likelihood ratio test for that variable was <0.05.

## Results

### Subjects

Longitudinal analysis was based on a group of 68 community-recruited normal subjects with two structural MR scans, diffusion MRI taken at the baseline date, and clinical data gathered at or near the dates of the structural MRIs. This group contained 12 subjects who later converted to MCI or AD, and 56 who remained stable. Demographics of the subjects are displayed in Table 1. Except for information on clinical conversion, all other data in the table reflect baseline date evaluations.

**Table 1:** **Demographics of normal cohort having DTI**.

Characteristic	Converters (*N* = 12)	Non-converters (*N* = 56)
Gender (M/F)	3/9	15/41
Age*	76.7 (7.2)	72.1 (6.9)
Education (years)	11.7 (3.6)	11.4 (5.1)
Time to convert (years)	2.2 (1.1)	–
MMSE	27.4 (3.1)	28.5 (1.6)
Episodic memory (*Z*-score)*	−0.48 (0.75)	0.20 (0.78)
Executive (*Z*-score)*	−0.35 (0.55)	0.08 (0.55)
ApoE4 (%)	25%	22%
Ethnicity (W/H/AA/other)	1/8/1/2	21/30/3/2

*For ethnicity designations: W means white, H means Hispanic, AA means African American. Starred variables are significantly different between converters and non-converters*.

### Longitudinal brain-tissue loss associated with baseline WM and GM ROIs

In the regression tests of ROI GM volume (including hippocampal volume) and voxelwise log-Jacobians, we found only one small significant cluster (2 cm^3^) log-Jacobian cluster for *t* > 3.5, corresponding to hippocampal volume. Other GM ROIs had no significant clusters.

**Figure 1:**
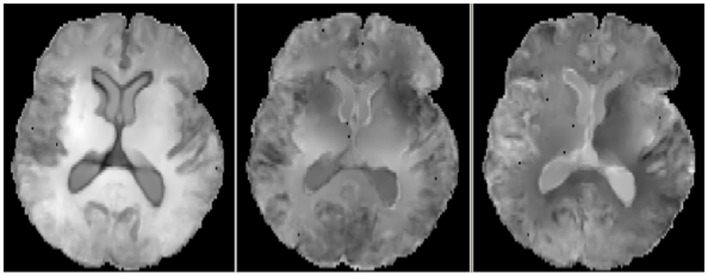
**Axial views of raw *t*-values from voxel regressions of log-Jacobians vs. FA**. Left: regressions with fornix body FA. Middle: regressions with splenium. Right: regressions with posterior cingulate. Bright indicates positive *t*-values, reflecting correlation of high ROI FA with smaller brain tissue loss.

**Figure 2:**
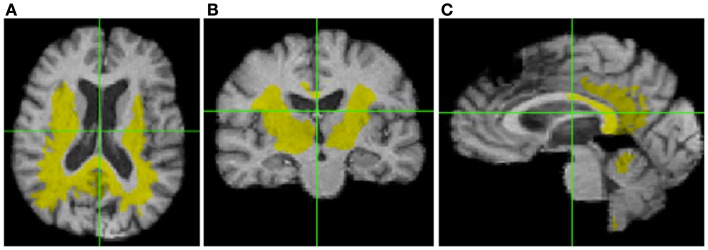
**Significant cluster of association (fSROI) between fornix body FA mean and negative log-Jacobians (*t* > 3.5)**. Views from left to right: **(A)** axial, **(B)** coronal, and **(C)** sagittal.

For the correlations with WM ROIs, Figure 1 displays raw *t*-values of log-Jacobians vs. FA in (left to right) the fornix body, splenium, and posterior cingulum. A consistent pattern of highly positive correlations is seen for the fornix (left panel) as compared to inconsistent and low magnitude associations for the other two ROIs in the middle and right panels. Sizes of significant clusters for all WM ROIs, corresponding to a threshold of *t* > 3.5, are given in Table 2. None of the anterior and posterior cingulate, the splenium, and genu ROIs had any significant clusters for *t*-values above this threshold. The CST had a very small cluster. The fornix body generated much larger ROI comprising components bilaterally in the posterior parietal, temporal, and frontal lobes, with small sections in the cerebellum. We will henceforth designate the ensemble of these clusters as *fornix significant ROI* (fSROI). Its tissue composition consisted of about 80% white and 20% gray matter. The fSROI is shown in Figure 2.

**Table 2:** **Volumes of significant clusters in regression of ROI FA values with voxel log-Jacobians, for *t* > 3.5**.

WM FA ROIs	Log-Jacobian significant cluster (cc brain-tissue loss) for *t* > 3.5
CST	0.76
Anterior cingulate	0
Posterior cingulate	0
CC genu	0
CC splenium	0
Fornix body	83.20

*There were no significant clusters for any GM ROI corresponding to threshold *t* > 3.5 except for the hippocampi, with cluster volume 2 cm^3^*.

### Anatomical composition of the fSROI

**Figure 3:**
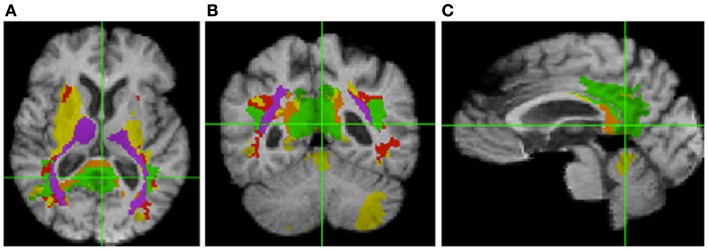
**Intersection of fSROI with ROIs in Table 3**. Warm colors: Tan, splenium, and projections from corpus callosum; red, longitudinal fasciculi. Cool colors: green, Brodmann areas; purple, thalamus and projections from thalamus. Yellow: fSROI regions not intersecting any of these ROIs. Left to right: **(A)** axial, **(B)** coronal, and **(C)** sagittal.

Table 3 shows the intersection of the fSROI with selected BA, regions of the corpus callosum, thalamus, longitudinal fasciculi, and WM tracts from the CC and thalamus to the parietal and occipital lobes. It indicates extensive overlap with the posterior cingulate cortex (PCC) (BA 23 and 31) and RSC (BA 26–29 and 30), as well as the thalamus. More generally, the fSROI heavily intersects the posterior portions of the corpus callosum, longitudinal fasciculi, and several of the WM tracts from thalamus or CC to posterior parietal or occipital gyri. Color-coded maps of intersections with ROIs listed in Table 3 are shown in Figure 3. Green areas illustrate extensive overlap of BA; purple shows overlap with thalamus and its projections; and tan shows coverage of the splenium and projections from the corpus callosum.

**Table 3:** **Component brain regions, which overlap with the whole fornix significant association ROI (fSROI)**.

(A)	
Brodmann ROIs	fSROI overlap (% of ROI)
BA 39 (angular gyrus)	20
BA 31 (dorsal PCC)	27
BA 30 (part of RSC)	41
BA 23 (ventral PCC)	61
BA 41 (auditory)	45
BA 26–29 (part of RSC)	78

**(B)**	
**Corpus callosum ROIs**	**fSROI overlap (% of ROI)**

CC MOG (middle occipital gyrus)	30
CC SOG (sup occipital gyrus)	43
CC SPG (sup parietal gyrus)	47
CC PrCU (precuneus)	50
CC Cu (cuneus)	55
Splenium	65

**(C)**	
**Thalamus ROIs**	**fSROI overlap (% of ROI)**

TH PrCu (precuneus)	51
Thalamus	51
TH SPG (sup parietal gyrus)	49
TH SOG (sup occipital gyrus)	58
TH MOG (middle occipital gyrus)	56

**(D)**	
**Longitudinal fasciculi ROIs**	**fSROI overlap (% of ROI)**

IFOF	22
ILF	22
SLF	34

*Right column shows what percentage of the given ROI is intersected by fSROI. (A) Brodmann areas intersecting fSROI. (B) Corpus callosum (CC) and tracts originating in CC. (C) Thalamus (TH) and tracts originating there. (D) Longitudinal fasciculi; CC_SOG, white matter tract from CC to superior occipital gyrus, etc.; IFOF, inferior frontal occipital fasciculus; TH_PrCu, thalamo-precuneus tract, etc.; ILF, inferior longitudinal fasciculus; SLF, superior longitudinal fasciculus; SPG, superior parietal gyrus (encompasses precuneus and connections from CC and thalamus); RSC, retrosplenial cortex and underlying white matter (encompasses Brodmann areas 26, 29, and 30)*.

### Brain factors as predictors of cognitive conversion

We tested the significance of the fSROI along with baseline brain measurements in Cox proportional hazards models for cognitive conversion from normal. Each model contained the multiple demographic variables described previously along with a single brain factor. Significant factors in the individual models, with their *p*-values and those of significant covariates, are summarized in Table 4. To facilitate effect comparisons amongst all brain variables and with age, hazard ratios for each brain factor are provided based on unit change in *Z*-score for brain factors and 1 year increase for age. Significant GM predictors of conversion were volumes in the ventral and dorsal entorhinal regions (BA 28, 34), the dorsal anterior cingulate (BA 32), and bilateral hippocampal volume. Age and ethnicity were also significant factors in the GM models. Fornix body FA and mean tissue change in fSROI were the other significant brain predictors of conversion. We note that in the GM models, age was highly significant and except for BA 28, it had a lower *p*-value than the accompanying brain factor. In the models of fornix FA and fSROI, by contrast, age was not significant.

**Table 4:** **Statistically significant factors for cognitive conversion (decline from normal) in Cox proportional hazards models**.

Brain factor in survival model	Brain factor *p*-value	Brain factor hazard ratio per unit *Z* increase	Age hazard ratio per year increase	Other significant factors (*p*-value)
BA 28 (ventral ER) GM	0.001*	0.34 (0.16–0.63)	1.15 (1.04–1.31)	Age (0.008), ethnicity (0.01)
BA 32 (dorsal AC) GM	0.03*	0.46 (0.23–0.92)	1.16 (1.04–1.32)	Age (0.006), ethnicity (0.02)
BA 34 (dorsal ER) GM	0.02*	0.47 (0.26–0.86)	1.17 (1.05–1.33)	Age (0.005), ethnicity (0.05)
Hippo volume	0.04*	0.47 (0.21–0.95)	1.22 (1.08–1.41)	Age (0.001), ethnicity (0.03)
Fornix body FA	0.015*	0.38 (0.16–0.83)	1.11 (1.00–1.26)	Ethnicity (0.037)
fSROI log-Jacobian	0.045*	0.45 (0.20–0.98)	1.11 (0.99–1.25)	Ethnicity (0.03)

*Each model contained age, gender, education, and ethnicity together with one brain factor. *p*-Values refer to the probability of the χ^2^ value being larger than computed for a given variable in likelihood ratio test. All brain factor values were converted to *Z*-scores prior to analysis in order to enable meaningful comparisons of hazard ratios. The hazard ratio columns show change in conversion risk per unit *Z*-score change (in brain factors) or year (age). Values in parentheses for these columns are the lower and upper 95% confidence interval endpoints. GM regions BA 26–29, 23, 31 are not shown because not significant; nor were the FA white matter regions in anterior and posterior cingulate, genu, and splenium; BA, Brodmann area; AC, anterior cingulate; ER, entorhinal cortex; fSROI, fornix significant ROI*.

### Interactions between GM, WM, and fSROI tissue loss

To test for confounding effects of age or other demographic factors in association between baseline fornix FA and fSROI log-Jacobians, we modeled the mean fSROI values as outcome of a multi-regression including independent variables of age, gender, education, ethnicity, and fornix FA. The overall model produced a strong fit (*R*^2^ = 0.52) with significance of the independent variables as follows: fornix FA, *p* = 0.0008; age, *p* = 0.016; education, *p* = 0.17; gender, *p* = 0.23; and ethnicity *p* = 0.69. Thus, fornix FA and age were the only significant correlates of tissue change in fSROI, with FA being much stronger than age.

To test for GM contributions to fSROI outcomes, we performed multiple regression modeling of mean fSROI log-Jacobians vs. baseline hippocampal, BA 28 or BA 34 gray volumes, one per regression model, together with the demographic factors of age, education, gender, and ethnicity. In each of these models, the overall fit was not as good as for the fornix FA (*R*^2^ was about 0.36 in each case) and no brain factor was significant, while age was significant in each model.

Finally, to test the mutual interaction of the brain variables, we examined the correlation matrix of components including the fSROI log-Jacobians, fornix FA, and GM volumes from BA 28, 34, and bilateral hippocampus. The matrix is given in Table 5.

**Table 5:** **Correlation matrix for factor interaction analysis of GM, fornix FA, and fSROI variables**.

	fSROI	Fornix FA	BA 28 GM	BA 34 GM	Hippo volume
fSROI	1.00	0.60	0.17	0.20	0.29
Fornix FA		1.00	0.38	0.45	0.31
BA 28 GM			1.00	0.58	0.35
BA 34 GM				1.00	0.38
Hippo volume					1.00

*From the table, fSROI is strongly correlated (0.60) with fornix FA but with no GM factor or the hippocampus. But fornix FA, while being most strongly correlated with fSROI, is also correlated with BA 28 and 34 (0.38 and 0.45, respectively). And BA 28 and 34 are strongly correlated with each other, and less so with hippocampal volume*.

## Discussion

In this study, an analysis of the relation between baseline ROI FA measurements and longitudinal brain change has indicated that fornix body microstructure is significantly associated with a large region of tissue loss (fSROI) primarily in the WM of the posterior frontal, parietal, and temporal lobes. Similar correlation analyses of WM in the CST, anterior and posterior cingulate, genu, and splenium, showed small or non-existent effects. Baseline GM values for the entorhinal cortex and hippocampus also failed to show strong associations with longitudinal brain change. The fornix has already been identified as a sensitive predictor of early cognitive change in normals (Oishi et al., 2012; Zhuang et al., 2012; Fletcher et al., 2013a) – a result corroborated in our present study – and our current findings now also link it with longitudinal change in posterior brain structures known to be affected by AD pathology (Table 3; Figure 3). Our regressions incorporating age as a covariate have indicated that these associations are not merely dependent on age. And the tissue losses in the fSROI are themselves significant predictors of conversion, in Cox survival models that included age as a covariate. The picture is nuanced, however, because GM regions in the limbic circuit also predicted cognitive conversion (Table 4) but not longitudinal brain change. In this section, we discuss these findings in relation to previous work, and attempt to draw some inferences about the nature and timing of early brain changes associated with cognitive decline.

### fSROI, limbic circuit, and prior results characterizing early AD

Table 3 and Figure 3 outline the intersection of the fSROI with other brain regions. The fSROI has relatively large overlaps with the thalamus; projections of the thalamus to precuneus; the superior parietal gyrus (including precuneus) and underlying WM; the RSC (BA 26, 29, and 30); the posterior cingulum (BA 23 and 31); and the splenium of the corpus callosum. Most of these are components of the circuit of Papez or limbic circuit (Nestor et al., 2003), considered to be important in the formation and consolidation of memory (Thomas et al., 2011). The fSROI is similar to the ROI found in a previous study investigating disconnection of the hippocampus and PCC in a cohort of early AD subjects (Villain et al., 2008). Their ROI of correlations between WM and hippocampal GM *Z*-scores (*p* < 0.05 uncorrected) included most of the cingulum bundle from frontal to parahippocampal fibers. They reported a correlation between gray matter *Z*-scores of the hippocampus and damage to the retrosplenial BA 29, 30, together with WM disruption of the posterior cingulum bundle. Cingulum bundle atrophy was in turn found to be correlated with glucose hypometabolism of the thalamus, mammillary bodies, middle cingulum, parahippocampal cingulum, and hippocampus – again components of the Papez circuit. The difference from our study was that in their early AD subjects the ROI arose from correlations of hippocampal and WM disruption, whereas our similar fSROI was correlated with fornix microstructural integrity at baseline. We shall discuss this below.

Another study (Acosta-Cabronero et al., 2010) examined the degeneration of WM tracts in early AD, using axial and radial diffusivities and mean diffusivity (MD). It found degeneration of tracts in the Papez circuit including the fornix, splenium, posterior cingulum, parahippocampal gyrus, and adjacent tracts of the temporo-parietal cortex as compared with normal controls. Anterior WM tracts were relatively spared. An important conclusion, according to that paper, is that this limbic circuit is preferentially vulnerable to degeneration in AD. This echoes an earlier proposal, based on the study of hypometabolism in MCI and AD subjects, that damage to the limbic circuit is the earliest significant event in the progression of AD (Nestor et al., 2003).

To summarize, then, previous studies both of hypometabolism and WM tract disruption among MCI and AD cohorts have found degeneration in regions which our fSROI extensively overlaps and in fact resembles. This is consistent with the possibility that the fSROI represents early longitudinal tissue changes, predicted by baseline fornix FA, which precede and eventually result in the effects described in those studies. This possibility is strengthened by our finding that brain-tissue losses in the fSROI are themselves significantly associated with cognitive conversion in survival models (Table 4).

### fSROI and the default mode network

Table 3 and Figure 3 show that the fSROI extensively overlaps the RSC. The RSC has been identified with the one of the main nodes, the PCC/RSC node (Greicius et al., 2009) of the DMN (Greicius et al., 2003), which is connected to a second principal node, the medial prefrontal cortex, by the cingulum bundles (Greicius et al., 2009). The RSC is also connected more ventrally to the medial temporal lobes probably by fibers in the descending (parahippocampal) cingulum (Thomas et al., 2011). In AD subjects, the DMN has diminished resting state activity in the PCC (Greicius et al., 2004), probably resulting from disrupted connectivity between the hippocampus and posterior cingulum, and DMN activity may be a sensitive biomarker for incipient AD. Further, among five distinct functional connectivity networks of the brain selectively targeted by differing neurodegenerative diseases, the DMN is the one specifically characterized by vulnerability to AD (Seeley et al., 2009). Because the fSROI extensively overlaps the RSC and posterior cingulum, it appears that the fSROI contains early tissue loss in principal posterior components of the DMN, thereby presaging the later loss of connectivity and decreased activity. And the strong association of the fSROI with fornix FA therefore suggests that fornix microstructure in cognitively normal subjects may be a very early marker of future disconnection in the DMN.

### Inferences about early brain processes of degeneration

The fornix and the rest of the limbic circuit, along with the DMN, are all selectively vulnerable to early Alzheimer’s pathology (Nestor et al., 2003; Seeley et al., 2009; Acosta-Cabronero et al., 2010). And degeneration in the limbic circuit has been proposed as the first significant event in the progression of AD (Nestor et al., 2003). If our identification of the fSROI with major components of the limbic circuit and DMN is correct, then we may have traced the trajectory of tissue losses in these circuits farther backwards toward their origins than has been done heretofore. In this early phase, the changes are associated with baseline fornix WM but not strongly with temporal gray matter. Tissue losses in fSROI are greater than due to aging alone, since their correlation with fornix FA was much stronger than with age. On the other hand, baseline GM measurements in the hippocampus and entorhinal cortices (also components of the limbic circuit) were significant predictors of cognitive decline but not of any brain-tissue loss. Analysis of these components (Table 5) showed that entorhinal areas were correlated with each other and with fornix FA but not strongly with fSROI. This may support the inference of two processes in early deterioration. One process may contribute most heavily to fornix WM and fSROI changes, underlying that strong correlation. The other may contribute to GM in the temporal lobe ROIs and also to fornix WM, but little to fSROI. Thus early deterioration in the posterior limbic circuit may be at least partly separate from gray matter processes in the entorhinal cortex or hippocampi. This inference does not necessarily contradict previous findings relating fornix and hippocampus (Lee et al., 2012) in MCI and AD, or cingulate WM and hippocampus (Villain et al., 2008, 2010) in early AD, because in the later stages, gray matter atrophy is more advanced and may contribute to circuit deterioration. Furthermore, a previous study has suggested (Sexton et al., 2010) that though fornix and gray matter pathologies may not be independent, the relationship may be complex, involving factors of Wallerian degeneration as well as separate WM disruptive processes. And another study (Douaud et al., 2013) has found that more than 2 years prior to conversion, WM microstructural differences in the fornix, left fimbria, and SLF characterized MCI patients who converted to probable dementia, when compared with other stable MCI subjects. Those findings appear to be consistent with the suggestions put forth here, that in the prodromal phase, fornix microstructure, and tissue loss in the limbic circuit overlain by fSROI may have a component independent of gray matter change.

### Limitations

We must note several limitations of our study. The first limitation is the small size of our normal cohort. This may have restricted our statistical power, for example in seeing relationships between GM ROIs and longitudinal brain change. The second limitation is the low resolution of our diffusion MRI data and the uncertainty of using it to make inferences about a structure as small as the fornix. It is evident from recent studies (Metzler-Baddeley et al., 2012; Berlot et al., 2014) that caution is required when interpreting diffusivity measurements, because of free water contamination to WM tracts generally and particularly in the fornix, due to its small size and proximity to the ventricles. But FWE (Pasternak et al., 2009) seems to improve FA’s distinctions between groups and the robustness of inferences about WM change (Metzler-Baddeley et al., 2012). In our experiments, FWE improved the statistical power of FA correlations with longitudinal brain change, enabling us to see a more clear contrast between the effects of the fornix and other WM tracts (Figure 1), while dampening diffusivities such as MD and their apparent statistical power (data not shown). Lastly, we were unable in this project to explore hypotheses about the possible origins of WM changes in the fornix and the associated tissue loss in the fSROI, and whether such changes are related to gray matter changes by some underlying mechanism. A likely candidate for such exploration would be brain levels of β-amyloid accumulation.

## Conclusion

In a cohort of normal subjects, baseline fornix WM microstructure significantly correlates with early longitudinal tissue change across an fSROI intersecting the posterior DMN and limbic circuits. These circuits are known to be selectively vulnerable to AD pathology. Baseline fornix WM microstructure, longitudinal tissue loss in the fSROI, and baseline GM volumes in the entorhinal cortices and hippocampus all are significantly associated with cognitive conversion from normal. But only fornix WM is associated with brain-tissue loss in the fSROI. Thus, WM deterioration in the fornix and tissue loss in fSROI may be the early beginnings of posterior limbic circuit and DMN degeneration. This early degeneration appears to be partly separate from gray matter atrophy. Future research may either identify separate mechanisms for early GM and WM change, or else show a common underlying process, which at the early stages manifests itself more strongly in the fornix–fSROI association.

## Conflict of Interest Statement

The authors declare that the research was conducted in the absence of any commercial or financial relationships that could be construed as a potential conflict of interest.
